# Declining Outdoor Recreation and Increased Use of Fitness Centers Among Norwegian Adolescents, 2010–2019

**DOI:** 10.3390/ijerph22081256

**Published:** 2025-08-11

**Authors:** Solveig Sandaker Liland, Vidar Sandsaunet Ulset

**Affiliations:** 1 Kvæfjord Municipality, Bygdeveien, 9475 Borkenes, Norway; 2 Department of Teacher Education and Outdoor Studies, Norwegian School of Sport Sciences, Sognsveien 220, 0863 Oslo, Norway; 3 PROMENTA Research Centre, Universitetet i Oslo, 1094 Blindern, 0317 Oslo, Norway

**Keywords:** outdoor recreation, fitness, population density, mental health, nature, Ungdata

## Abstract

Adolescents’ physical activity patterns appear to be changing, with indications of a shift from nature-based activities toward more structured, indoor forms of exercise. However, it remains unclear how participation in outdoor recreation and fitness center use has developed in parallel over time, and whether these trends vary by degree of urbanization. The aim of the present study was to examine the trends in adolescents’ engagement in outdoor recreation and use of fitness centers across Norwegian municipalities between 2010 and 2019 and to assess how these patterns relate to individual and contextual factors. Repeated cross-sectional data were drawn from the Ungdata survey (N = 67,554), and multilevel linear models were applied to estimate time trends and test interactions with municipal population density. Analyses were adjusted for depressive symptoms, gender, school grade, and vegetation density (NDVI). The results indicated a significant decline in outdoor recreation during the period, particularly in more urban municipalities, alongside a marked increase in fitness center use. The two activity types were positively associated but not mutually exclusive. The findings point to a broader shift in adolescent activity preferences that may reflect changing environmental and sociocultural conditions. These patterns underline the need for public health approaches that recognize diverse forms of youth engagement in physical activity.

## 1. Introduction

Urbanization has significantly reshaped the landscapes in which today’s adolescents grow up, altering their physical, social, and psychological environments. According to global projections, 68% of the world’s population will reside in urban areas by 2050, up from 55% in 2019 [1]. Norway is already ahead of this trend, with more than 80% of its population living in densely populated areas [2]. With this shift comes greater distance from natural surroundings and increased exposure to built environments, potentially limiting opportunities for outdoor play and physical activity [3]. At the same time, advances in technology and the pervasive use of digital media have transformed the ways in which young people socialize, entertain themselves, and engage with the world around them [4]. In parallel with these trends, there has been an increase in mental health problems in youth, especially during the last decade [5]. This raises concerns about a growing disconnection between adolescents and nature, as well as the changing patterns of physical activity with a shift away from unstructured outdoor play toward more structured indoor environments, such as fitness centers. While these forms of activity offer different opportunities and experiences, understanding how they evolve over time is important for supporting diverse modes of engagement in physical activity.

Outdoor recreation holds a central place in Norwegian identity and is recognized by national authorities as a key public health strategy [6]. A growing body of interdisciplinary research highlights the physiological and psychological benefits of nature exposure. The Biophilia hypothesis posits an innate human affinity for nature [7], while the Stress Reduction Theory [8] and Attention Restoration Theory [9] argue that natural environments promote emotional regulation and cognitive recovery. These perspectives are supported by empirical studies showing that time spent in nature is associated with reduced stress [10], improved mood [11], and enhanced mental functioning [12,13,14], particularly among children and adolescents. In this context, access to nature is not merely a recreational asset but a foundational element of healthy development.

Fitness centers offer structured, indoor environments for physical activity that may align more closely with contemporary youth culture, especially in urban contexts. Research has found that fitness center use among adolescents can have both positive and negative implications. Regular physical activity and cardiovascular fitness are associated with a range of positive outcomes in adolescents, including reduction in chronic diseases [15], better mental health [16], and improved cognitive functioning [17]. Fitness center attendance specifically has also been linked to well-being and mental health [18]. Recent research indicates that the motives for fitness center use among adolescents often emphasize appearance and enjoyment, with body-related goals being particularly salient among younger users [19]. As such, fitness centers may emphasize efficiency, performance, and physical appearance—characteristics that align with current societal norms but may also foster appearance-related pressures among youth. For example, one study found that time spent exercising within fitness centers was more strongly associated with negative body image and disordered eating symptoms than exercise performed outside of the fitness center environment, particularly when driven by appearance-focused motives [20]. These observations underscore the need to better understand how adolescents’ engagement with fitness centers is developing over time and how this relates to shifts in outdoor recreation and broader environmental and societal factors relevant to youth physical activity.

Over recent decades, the everyday lives of youth have become more structured and scheduled, often at the expense of unstructured outdoor time. Participation in self-organized outdoor play has declined, replaced by organized sports, screen-based activities, and school-centered routines [21]. These changes have occurred alongside an increase in mental health challenges among adolescents, particularly related to anxiety and depression [5,22]. Moreover, it has been hypothesized that traditional outdoor activities may be losing ground to indoor exercise environments, such as gyms and fitness centers, particularly in urban areas where such facilities are more accessible and culturally promoted [23]. This trend may not only reflect reduced access to or engagement with natural environments but also broader sociocultural shifts in how adolescents socialize, pursue physical activity, and express identity in structured, performance-oriented contexts. Such changes may also be adaptive, particularly in light of increased urbanization and the need for accessible, structured arenas for physical activity and social belonging.

While several earlier studies have identified a decline in outdoor activity among adolescents [24,25,26], no studies have systematically examined how these trends vary across municipalities with different levels of population density. Although most prior research has focused on single-year snapshots or limited cohorts, the present study contributes by providing updated, population-based analyses covering the years leading up to the COVID-19 pandemic. Establishing a robust pre-pandemic baseline is crucial, as the pandemic may have introduced lasting shifts in youth activity patterns. By focusing on population density as a key contextual moderator, this study helps illuminate how broader structural conditions, such as urbanization, may influence adolescents’ engagement in outdoor recreation versus indoor exercise. This contributes to a more nuanced understanding of how individual behavior is shaped not only by personal factors but also by the characteristics of the environments in which young people live.

The present study analyzes national survey data from Ungdata (2010 to 2019) [27] to investigate how adolescents’ participation in two forms of physical activity, outdoor recreation and use of fitness centers, has developed over time. The analysis focuses on identifying trends in reported activity and examines whether these patterns differ by municipal population density. In addition, the study assesses how demographic and environmental factors such as depressive symptoms, gender, grade level (as a proxy for age), and local vegetation density are associated with activity levels. By considering both outdoor and indoor exercise settings, the study aims to contribute a more differentiated understanding of trends in adolescents’ physical activity behaviors within changing environmental and societal contexts.

## 2. Materials and Methods

### 2.1. Data Source: Ungdata Survey

This study draws on data from the national Ungdata survey, a repeated cross-sectional and standardized self-report questionnaire offered to municipalities and counties across Norway. Initiated in 2010, Ungdata has since 2014 been administered in both lower and upper secondary schools, with students completing the survey during school hours. With response rates exceeding 80% in lower secondary and slightly lower in upper secondary, Ungdata provides broad population coverage. The survey includes core modules that all participants complete, as well as optional modules selected by participating municipalities. The survey is anonymous, digital, and designed to capture a wide range of youth experiences, including physical activity, mental health, and leisure behavior. Because students respond only once, the dataset enables cross-sectional analyses of national and regional trends across cohorts.

### 2.2. Sample

The original Ungdata dataset includes responses from 628,678 adolescents. For the present study, the analysis focused on the subset of participants who completed both the core module and the optional Module E: Leisure Time, which contains items related to outdoor recreation. The selection of optional modules in Ungdata is determined at the municipal level in collaboration with the regional drug and alcohol competence centers (KoRus). This means that not all municipalities include the same thematic modules in every survey year. The final analytic sample comprised 67,554 adolescents from lower and upper secondary schools who responded to relevant items between 2010 and 2019. These participants were distributed across 100 municipalities and 15 counties. Previous evaluations have confirmed that Ungdata is nationally representative and that annual variation in participating municipalities and selected modules does not substantially bias national trend estimates [27].

### 2.3. Measures

Outdoor Recreation. Outdoor recreation was assessed via Ungdata Module E.14 [28], which asked students how frequently they participated in a range of seasonal outdoor activities. These included (1) cross-country skiing in forests or mountains, (2) hiking, (3) skateboarding, (4) snowboarding or alpine skiing, (5) fishing, (6) overnight camping in nature (excluding organized campsites), (7) swimming, (8) canoeing or kayaking, and (9) climbing. Response options ranged from “several times a week” to “never/almost never,” on a five-point scale.

Use of Fitness Center. Fitness center participation was assessed using the following item: *“How often do you participate in the following activities?—go to the gym or fitness center”*. Response options were (1) never, (2) rarely, (3) 1–2 times a month, (4) 1–2 times a week, (5) 3–4 times a week, and (6) at least 5 times a week. This item captures unorganized physical activity conducted individually or with friends in structured indoor environments, such as gyms or fitness centers, and is distinct from organized participation in sports clubs [28,29].

Demographics. Gender and grade level were included as background variables. Gender was coded as a binary variable, where 1 = boy and 2 = girl. Grade level served as a proxy for age and was categorized as lower secondary (8th–10th grade) or upper secondary (VG1–VG3). To improve data quality, grade-level response options were restricted after 2014 to reflect students’ actual school level and prevent implausible combinations of age and grade.

Depressive Symptoms. Symptoms of depression were measured using a six-item short form from the Hopkins Symptom Checklist [30]. Students rated how often during the past week they had experienced (1) everything felt like a struggle, (2) trouble sleeping, (3) feeling unhappy or depressed, (4) hopelessness about the future, (5) feeling tense or stiff, and (6) excessive worry. Responses were given on a four-point scale ranging from “not at all” to “very much.” The resulting composite score reflects subclinical depressive symptoms.

Municipality-Level Variables. Municipal identifiers allowed us to link survey data to contextual indicators at the municipal level. Two municipality-level variables were included in the analysis: population density (measured as residents per square kilometer) and the normalized difference vegetation index (NDVI) [31]. NDVI quantifies vegetation cover based on satellite imagery and served as a proxy for green space exposure. Values were derived from the MODIS/Terra Vegetation Indices dataset (MOD13Q1, Version 6.1), which provides 250 m spatial resolution and 16-day temporal resolution. To ensure comparability and reduce bias from cloud interference, the median NDVI for each municipality was calculated across the 12-month period from 1 January 2019, to 1 January 2020. NDVI has been validated as a robust indicator of residential green space exposure [32,33]. Municipality classification followed the standard national schema for administrative regions as of 2019 [34].

### 2.4. Statistical Analyses

To examine trends in participation in outdoor recreation and fitness center training from 2010 to 2019, multilevel linear regression analyses were conducted in R (version 4.4.2), using the lme4 package [35]. Two-level hierarchical models were specified, with individuals nested within municipalities. Outdoor recreation and fitness center training were modeled separately. Year (centered at 2009) was included as a continuous predictor to capture linear trends over time and was the primary temporal variable of interest. Random intercepts were specified at the municipal level to account for clustering. To test whether trends over time differed by contextual factors, an interaction term between year and municipal population density (persons per km^2^) was included, which served as a proxy for level of urbanization. To account for potential behavioral substitution or complementarity, fitness center training was included as a predictor in the outdoor recreation model, and outdoor recreation was included in the fitness center training model. Depressive symptoms, gender, age (school grade), and NDVI were included as control variables to adjust for individual and environmental covariates not central to the hypotheses. All continuous predictors were standardized prior to analysis. In sensitivity analyses, models with quadratic time trends were examined to capture potential nonlinear patterns, and additional models were estimated with and without mutual adjustment for participation in outdoor recreation and fitness center use to assess potential behavioral overlap. Model fit was evaluated using marginal and conditional R^2^ values.

## 3. Results

### 3.1. Descriptive Trends

**Figure 1:**
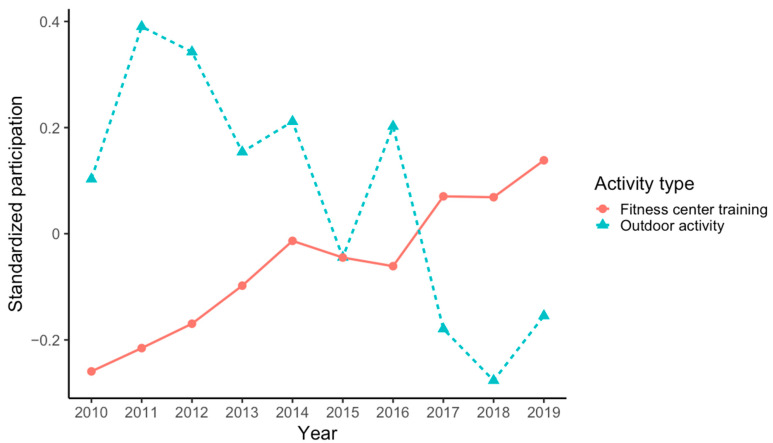
Trends in standardized participation in outdoor activity and fitness center training from 2010 to 2019. Lines represent the yearly average standardized score for self-reported engagement in outdoor activity (e.g., hiking, skiing, camping) and fitness center training (one item). Scores are based on z-transformed means per year, allowing comparison of relative change over time. Both activities show opposite trends, with outdoor activity decreasing and fitness center training increasing across the observed period.

**Figure 2:**
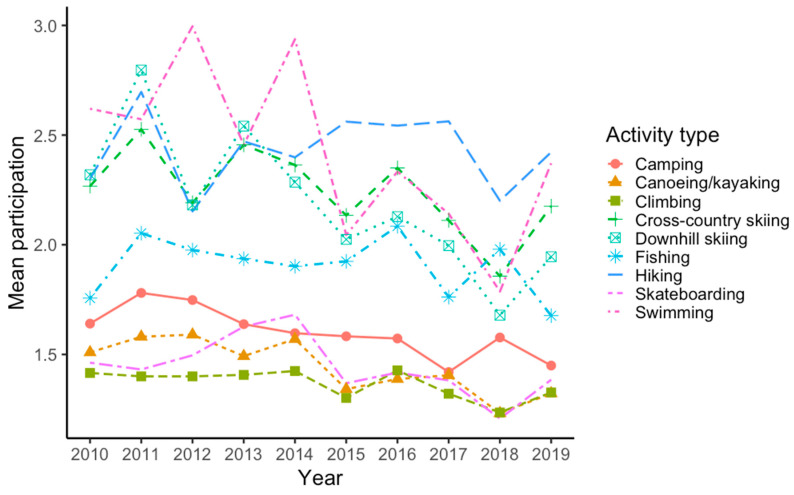
Trends in raw mean participation in specific outdoor activities from 2010 to 2019. The figure displays average reported engagement in nine types of outdoor recreation. Participation scores reflect raw item means per activity on a Likert scale, with higher values indicating more frequent participation. Each line represents the yearly average for one activity, with points marking annual means. Shaded areas are not shown; values are unadjusted and descriptive.

Figure 1 presents descriptive trends in standardized participation scores for overall outdoor activity and fitness center training from 2010 to 2019. These scores reflect raw, unadjusted means across years. A clear divergence is evident: participation in outdoor activity shows a gradual and consistent decline, while fitness center training increases steadily over time. Figure 2 displays descriptive trends for specific outdoor activities. Hiking and cross-country skiing consistently show the highest participation levels, though both exhibit some decline over time. Swimming and downhill skiing also show relatively high levels of participation across several years. Activities such as fishing and camping are moderately popular but fluctuate more across time. Climbing and skateboarding remain the least commonly reported activities throughout the period.

### 3.2. Multilevel Regression

Multilevel linear regression models were used to examine changes in outdoor recreation and fitness center training from 2010 to 2019, accounting for both individual- and municipal-level covariates. The results, presented in Table 1, reveal contrasting trends over time: outdoor recreation declined steadily, while fitness center training increased. These patterns varied by municipal population density, with steeper declines in outdoor activity and slightly stronger increases in fitness center use observed in urban areas. Additional predictors such as age, gender, depressive symptoms, and environmental context (e.g., vegetation density) were also associated with participation, and the two activity types were positively related but captured distinct behavioral patterns.

#### 3.2.1. Outdoor Recreation

**Figure 3:**
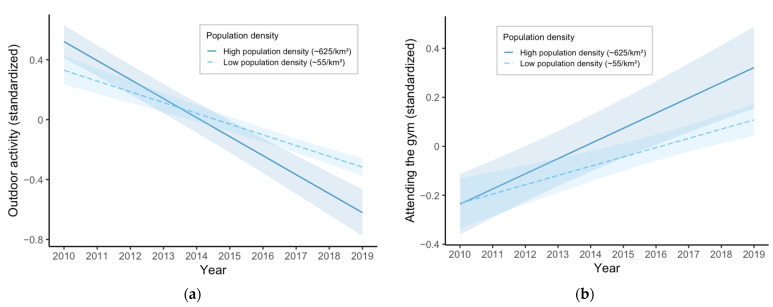
Trends in physical activity from 2010 to 2019 by municipal population density. (**a**) Predicted levels of outdoor recreation over time, moderated by population density; (**b**) Predicted levels of fitness center training over time, moderated by population density. Shaded areas represent 95% confidence intervals. Population density was operationalized as residents per square kilometer and categorized as low (~55/km^2^) or high (~625/km^2^).

The model predicting outdoor recreation showed a significant negative linear time trend, indicating a steady decline in outdoor activity across the study period (*β* = −0.12, 95% CI [−0.13, −0.10], *p* < 0.001). This suggests that, on average, participants reported lower levels of outdoor recreation in later years, independent of other variables in the model. Importantly, the interaction between year and municipal population density was significant (*β* = −0.06, 95% CI [−0.08, −0.03], *p* < 0.001), indicating that the decline in outdoor activity was more pronounced in urban municipalities (see Figure 3a). Among the covariates, depressive symptoms were negatively associated with outdoor recreation (*β* = −0.07, 95% CI [−0.08, −0.06], *p* < 0.001), indicating that participants who reported more depressive symptoms also reported lower engagement in outdoor activities. Gender was a significant predictor, with girls reporting slightly lower participation than boys (*β* = −0.04, 95% CI [−0.05, −0.03], *p* < 0.001). Grade level (used as a proxy for age) was negatively associated with outdoor recreation (*β* = −0.12, 95% CI [−0.13, −0.11], *p* < 0.001), suggesting that outdoor recreation tends to decrease as students progress through school. At the municipal level, vegetation density (NDVI) was positively associated with outdoor activity (*β* = 0.06, 95% CI [0.02, 0.09], *p* = 0.001), indicating that individuals living in greener municipalities were more likely to engage in outdoor recreation. Interestingly, population density was also positively associated with outdoor recreation (*β* = 0.25, 95% CI [0.10, 0.40], *p* = 0.001); however, the negative interaction with time suggests this relationship may change over the years. Finally, fitness center training was positively associated with outdoor activity (*β* = 0.09, 95% CI [0.08, 0.10], *p* < 0.001).

#### 3.2.2. Fitness Center Training

The model predicting fitness center training showed an opposite trend: a significant increase in participation over time (*β* = 0.06, 95% CI [0.04, 0.07], *p* < 0.001). Although the interaction between year and population density was only marginally significant (*β* = 0.02, 95% CI [−0.00, 0.05], *p* = 0.063), it suggests a potential trend in which the increase in fitness center training is somewhat more pronounced in more urban municipalities (see Figure 3b). Consistent with the outdoor recreation model, depressive symptoms showed a positive association with fitness center training (*β* = 0.02, 95% CI [0.01, 0.03], *p* < 0.001), suggesting that individuals reporting more depressive symptoms were slightly more likely to attend the fitness center. Gender was also a significant predictor in the same direction as for outdoor recreation, with girls reporting lower levels of fitness center participation (*β* = −0.05, 95% CI [−0.06, −0.04], *p* < 0.001). Grade level (as a proxy for age) showed a strong positive association with fitness center training (*β* = 0.30, 95% CI [0.29, 0.31], *p* < 0.001), indicating that fitness center training increases substantially with age. In contrast to the outdoor model, vegetation density had no significant association with fitness center participation (*β* = −0.00, *p* = 0.904), and municipal population density was not a significant predictor either (*β* = −0.03, *p* = 0.737). Finally, outdoor recreation was positively associated with fitness center training (*β* = 0.09, 95% CI [0.08, 0.09], *p* < 0.001).

#### 3.2.3. Sensitivity Analyses

To examine the extent to which participation in outdoor recreation and use of fitness centers overlapped, sensitivity analyses were conducted by estimating each multilevel model with and without adjustment for the other activity type. In the model predicting outdoor recreation, excluding the use of fitness centers as a covariate had minimal impact on the estimated time trend (β = −0.12 vs. −0.11). The explanatory power of the model decreased only slightly (marginal R^2^ from 0.100 to 0.090), indicating that the observed decline in outdoor activity over time remained robust regardless of adjustment for the use of fitness centers. In the model predicting the use of fitness centers, excluding outdoor recreation resulted in a modest attenuation of the time trend (β = 0.06 vs. 0.05) and a reduction in explanatory power (marginal R^2^ from 0.106 to 0.097). Thus, mutual adjustment had limited influence on model estimates, indicating shared but non-redundant variance between outdoor recreation and fitness center use.

## 4. Discussion

### 4.1. Main Findings

This study investigated national trends in adolescents’ engagement in outdoor recreation and fitness center training from 2010 to 2019. A significant decline in outdoor recreational activity was observed over the decade, paralleled by a marked increase in the use of fitness centers. These findings are consistent with prior national reports and studies indicating reduced participation in nature-based activities and a shift toward more structured or indoor exercise settings [24,25,26,36]. Our results suggest that this trend has continued beyond the time frames of earlier studies, underscoring the need for sustained attention to adolescent engagement with nature. The divergent trends in outdoor and gym-based activities may reflect broader cultural and structural shifts. Adolescents today are exposed to more time constraints, academic demands, and technology-based social interaction than previous generations [4]. Fitness center training may offer a more time-efficient and socially acceptable mode of physical activity, particularly in urban settings. Moreover, fitness and body image pressures may further motivate indoor training [20,37], while traditional outdoor activities may be perceived as less relevant or accessible. Importantly, fitness center training and outdoor recreation were not mutually exclusive. Adolescents engaging in one activity were often active in the other as well. However, our findings also point to the existence of adolescents who may disengage from both types of physical activity, aligning with prior research showing dropout in organized activity during adolescence [38].

### 4.2. Population Density as Moderator

One of the novel contributions of this study is the analysis of population density as a moderator of change. Our findings showed that the decline in outdoor recreation was more pronounced in municipalities with higher population density, whereas the increase in fitness center training tended to be stronger in those same areas. These results suggest that urban environments may offer fewer opportunities or reduced motivation for outdoor recreation, possibly due to lower availability or perceived accessibility of green areas, as well as increased competition from other indoor and commercial activities. Although many urban adolescents do have access to green spaces, these areas may not always be perceived as attractive, safe, or culturally valued spaces for recreation [39]. In contrast, fitness center facilities may be more visible, structured, and marketed toward youth, particularly in densely populated regions [23]. This underscores the need for place-sensitive strategies to support outdoor activity.

### 4.3. Associations of Outdoor Recreation and Fitness Center Training with Individual Characteristics

Consistent with earlier studies, depressive symptoms were negatively associated with participation in both outdoor activity and fitness center training [29]. Whether this reflects the activity’s protective effects or lower engagement due to poor mental health cannot be determined from these data, but the associations highlight the need to consider mental health as both an outcome and a determinant of physical activity engagement. We also observed small but significant gender differences: boys participated more in outdoor recreation and fitness center training than girls. Age, approximated by school grade level, was negatively associated with outdoor recreation and positively with fitness center training. This pattern suggests a developmental shift in adolescents’ preferences or availability for different activity types as they mature.

### 4.4. Strengths and Limitations

A key strength of this study is the integration of national self-report survey data with high-quality, municipality-level register data from Statistics Norway (SSB), including population density and NDVI (Normalized Difference Vegetation Index). This allowed to account for both individual and structural factors in modeling adolescents’ activity patterns. The use of repeated cross-sectional data across a ten-year span and in 75 municipalities enhances the generalizability and robustness of our findings. However, limitations must also be acknowledged. The reliance on self-reported activity introduces possible bias due to recall error or social desirability. The available items also limit precision: for example, fitness center attendance was assessed through a single frequency item, and emerging outdoor activities such as mountain biking or freeride skiing may not be well captured in the listed options. NDVI was measured only for the year 2019–2020 and used as a proxy for green space availability throughout the 2010–2019 period. This static measure does not capture potential changes in vegetation cover or urban development over time, which may have influenced adolescents’ access to green spaces during the study period. NDVI values may also be affected by year-specific weather conditions, such as precipitation, drought, or snow cover, which could in turn influence both vegetation indices and adolescents’ engagement in seasonal outdoor activities, introducing a potential source of confounding. The measure of outdoor recreation may not have captured emerging or urban forms of nature-based activity (e.g., city biking, parkour, or the use of urban green spaces), potentially underestimating adolescents’ engagement in alternative outdoor practices. Depressive symptoms were included as a psychological indicator due to consistent evidence of increasing mental health problems among adolescents in recent years [5]. While this measure highlights negative aspects of well-being, positive indicators, such as life satisfaction, could have provided a more balanced perspective. Additionally, the data are cross-sectional at each time point, limiting our ability to make inferences about individual-level change.

### 4.5. Implications and Future Directions

This study highlights the importance of understanding and addressing the changing patterns of physical activity among adolescents, particularly in urban areas. While participation in fitness center training appears to be increasing, the access to and engagement with outdoor environments is declining in many contexts. Both activity forms offer distinct opportunities: fitness centers may provide structured, accessible, and socially relevant spaces for exercise, especially in densely populated areas, whereas contact with natural environments has been associated with unique psychological and physiological benefits. A sustained decline in outdoor recreation could have long-term implications—not only for individual health and well-being but also for environmental connection and stewardship. To support a broad spectrum of health-promoting activities, policies and interventions should aim to preserve and improve access to green spaces while also recognizing the role of structured indoor environments in adolescent physical activity. Future research should examine how individual, social, and environmental factors shape young people’s activity preferences and how different forms of engagement can be supported through public health and education strategies.

## 5. Conclusions

This study examined the changes in adolescents’ participation in outdoor recreation and fitness center training in Norway from 2010 to 2019. The results showed a decline in outdoor activity and a parallel increase in fitness center use, particularly in urban municipalities. These opposing trends suggest a shift in adolescent activity preferences, potentially shaped by social, environmental, and developmental factors. While fitness center training may offer structured and accessible opportunities for physical activity, especially in dense urban areas, the decline in nature-based activities remains a concern given their known benefits for mental and physical health. The findings emphasize the need for policy and practice to support a wide range of physical activity options for youth. Preserving green spaces and fostering positive experiences with nature in childhood may be important for long-term health and environmental engagement. At the same time, the growth in fitness center use may reflect positive developments in health awareness and exercise culture. Future research should explore how structural conditions and psychological factors interact to shape youth activity patterns and how inclusive and sustainable opportunities for both indoor and outdoor activity can be promoted.

## Figures and Tables

**Table 1:** Multilevel models predicting outdoor recreation and fitness center training (standardized outcomes).

	Outdoor Recreation			Use of Fitness Center		
Predictors	Estimates	CI	*p*	Estimates	CI	*p*
Intercept (baseline level)	0.60	0.50–0.70	<0.001	−0.29	−0.41–−0.18	<0.001
Depressive symptoms	−0.07	−0.08–−0.06	<0.001	0.02	0.01–0.03	<0.001
Gender (1 = boy, 2 = girl)	−0.04	−0.05–−0.03	<0.001	−0.05	−0.06–−0.04	<0.001
Grade level (proxy for age)	−0.12	−0.13–−0.11	<0.001	0.30	0.29–0.31	<0.001
Vegetation density (NDVI)	0.06	0.02–0.09	0.001	−0.00	−0.04–0.04	0.904
Use of fitness center	0.09	0.08–0.10	<0.001			
Population density	0.25	0.10–0.40	0.001	−0.03	−0.19–0.13	0.737
Year (centered at 2010)	−0.12	−0.13–−0.10	<0.001	0.06	0.04–0.07	<0.001
Interaction: Year × Population density	−0.06	−0.08–−0.03	<0.001	0.02	−0.00–0.05	0.063
Outdoor recreation				0.09	0.08–0.10	<0.001
**Random Effects**						
σ^2^	0.92			0.90		
τ_00 (municipality)_	0.02			0.03		
ICC	0.02			0.03		
N _(municipality)_	75			75		
Observations	45,627			45,627		
Marginal R^2^/Conditional R^2^	0.100/0.120			0.106/0.134		

## Data Availability

The data and materials from the Ungdata Surveys are closed and stored in a national database administered by NOVA. The data are available for research purposes upon application. For requesting the data, please contact ungdata@oslomet.no. Further information about the study and the questionnaires can be found on the web page (https://www.ungdata.no/english/, accessed on 1 June 2025).
